# Mesenchymal Stem Cell-Derived Exosomes Ameliorate Alzheimer’s Disease Pathology and Improve Cognitive Deficits

**DOI:** 10.3390/biomedicines9060594

**Published:** 2021-05-24

**Authors:** Yi-An Chen, Cheng-Hsiu Lu, Chien-Chih Ke, Sain-Jhih Chiu, Fong-Shya Jeng, Chi-Wei Chang, Bang-Hung Yang, Ren-Shyan Liu

**Affiliations:** 1 Institute of Clinical Medicine, National Yang Ming Chiao Tung University, Taipei 112, Taiwan; yachen0414@gmail.com; 2 Molecular and Genetic Imaging Core/Taiwan Mouse Clinic, National Comprehensive Mouse Phenotyping and Drug Testing Center, Taipei 112, Taiwan; rocket2350@yahoo.com.tw (C.-H.L.); caesar1227@gmail.com (S.-J.C.); jengfs@gmail.com (F.-S.J.); 3 Industrial Ph.D Program of Biomedical Science and Engineering, National Yang Ming Chiao Tung University, Taipei 112, Taiwan; 4 Department of Medical Imaging and Radiological Sciences, Kaohsiung Medical University, Kaohsiung 807, Taiwan; 5 Drug Development and Value Creation Research Center, Kaohsiung Medical University, Kaohsiung 807, Taiwan; 6 Department of Medical Research, Kaohsiung Medical University Hospital, Kaohsiung 807, Taiwan; 7 National PET and Cyclotron Center (NPCC), Department of Nuclear Medicine, Taipei Veterans General Hospital, Taipei 112, Taiwan; cwchang@vghtpe.gov.tw (C.-W.C.); bhyang@vghtpe.gov.tw (B.-H.Y.); 8 Department of Biomedical Imaging and Radiological Sciences, National Yang Ming Chiao Tung University, Taipei 112, Taiwan; 9 Department of Nuclear Medicine, Cheng Hsin General Hospital, Taipei 112, Taiwan

**Keywords:** Alzheimer’s disease, exosome, mesenchymal stem cell, cell-free therapy, ^18^F-FDG

## Abstract

The accumulation of extracellular β-amyloid (Aβ) plaques within the brain is unique to Alzheimer’s disease (AD) and thought to induce synaptic deficits and neuronal loss. Optimal therapies should tackle the core AD pathophysiology and prevent the decline in memory and cognitive functions. This study aimed to evaluate the therapeutic performance of mesenchymal stem cell-derived exosomes (MSC-exosomes), which are secreted membranous elements encapsulating a variety of MSC factors, on AD. A human neural cell culture model with familial AD (FAD) mutations was established and co-cultured with purified MSC-exosomes. 2-[^18^F]Fluoro-2-deoxy-d-glucose ([^18^F]FDG) and novel object recognition (NOR) testing were performed before/after treatment to evaluate the therapeutic effect in vivo. The AD-related pathology and the expression of neuronal memory/synaptic plasticity-related genes were also evaluated. The results showed that MSC-exosomes reduced Aβ expression and restored the expression of neuronal memory/synaptic plasticity-related genes in the cell model. [^18^F]FDG-PET imaging and cognitive assessment revealed a significant improvement in brain glucose metabolism and cognitive function in AD transgenic mice. The phase of neurons and astrocytes in the brain of AD mice were also found to be regulated after treatment with MSC-exosomes. Our study demonstrates the therapeutic mechanism of MSC-exosomes and provides an alternative therapeutic strategy based on cell-free MSC-exosomes for the treatment of AD.

## 1. Introduction

Alzheimer’s disease (AD) is a progressive neurodegenerative disorder that accounts for 50–70% of the cases of dementia [1]. By 2025, the worldwide prevalence of AD will increase to 100 million or more, leading to a significant financial burden and augmenting the need of a high level of care. Clinical symptoms of AD include cognitive decline, short-term memory failure, orientation problems, and motor abnormalities [2,3]. These deficits are attributed to widespread neuronal loss, glial dysfunction, synaptic degeneration and brain atrophy, and gradual propagation of hallmarks of AD, including amyloid plaques and neurofibrillary tangles (NFTs), throughout the brain [4]. Amyloid plaques, a unique signature of AD, are deposits of excess aggregated amyloid-β (Aβ) peptides that are considered to initiate the pathogenic cascade that eventually leads to AD [5]. Various therapeutic approaches targeting Aβs have been explored, including the inhibition of either β-secretase or γ-secretase and immunotherapy; however, most of them did not exhibit positive outcomes in their phase III trials [6,7,8,9,10,11]. So far, FDA has approved only five medications for AD, including three cholinesterase inhibitors, one N-methyl-d-aspartate (NMDA) receptor antagonist, and a combination that targets the cholinergic and glutamatergic systems simultaneously [12]. These drugs may alleviate the symptoms of AD but do not slow down the progression of the disease. During the long process of Aβ aggregation, more than one physiological pathway crosstalk with each other to induce AD pathology [13]. Owing to the complicated pathological components, a single remedy targeting Aβs might not be able to cure AD. It is suggested that therapeutic targets for AD should include multiple strategies or combinational remedies for the maximum effectiveness.

The neuroprotective paracrine effects of mesenchymal stem cells (MSCs) are able to stimulate proliferation, neuronal differentiation, and survival in endogenous neurogenic niches in cellular models of AD [14,15]. In rodent AD models, MSC transplantation has been reported to reduce Aβ deposition, stimulate neurogenesis, improve spatial learning and memory deficits [16]. AD, as a neurodegenerative disorder, is characterized by mass neuronal and synaptic loss; therefore, regeneration of neuronal circuits by exogenous MSCs is a rational therapeutic strategy [17]. Nevertheless, the risks of tumor formation, immune rejection, and infusional toxicity in MSC transplantation remain unresolved. Increasing evidence suggests that MSC-derived exosomes (MSC-exosomes) as a cell-free therapy is equally effective as MSCs; in addition, MSC-exosomes do not pose the risk of tumorigenesis, are easier to produce and store, and are even less immunogenic than their parent cells [18,19]. Exosomes, nanometer-sized membrane vesicles (50–200 nm), can transfer endogenous proteins, lipids, mRNAs, and miRNAs into recipient cells where their components can reprogram the molecular machinery. Exosomes are naturally secreted by a variety of cells when multivesicular endosomes fuse with the plasma membrane and further mediate local or systemic cell-to-cell communication [20,21,22]. Exosomes could be facilitated in cross the blood–brain barrier (BBB) during stressful states, such as inflammatory conditions [23,24,25]. Administration of MSC-exosomes can promote neurogenesis in the subventricular zone (SVZ) and dentate gyrus (DG) of the hippocampus in different mice models of disease including AD and augment neuroprotection against inflammation and oxidative stress [25,26,27]. Thus, the comprehensive therapeutic effect of MSC-exosomes on AD is worthy of pursuit.

Herein, we generated a FAD human neural cell culture model that mimicked AD pathology by overexpressing human APP with both Swedish (K670N/M671L) and London (V717I) FAD mutations in a human neuroblastoma cell line to investigate the therapeutic effect of MSC-exosomes in vitro [28]. The expression of Aβ and of several neuronal memory/synaptic plasticity-related genes was evaluated after MSC-exosomes treatment. The glucose analog 2-[^18^F]fluoro-2-deoxy-d-glucose ([^18^F]FDG) is the most frequently used radiotracer to measure cerebral glucose metabolism for the diagnosis of AD in patients. The reduction of brain glucose metabolism correlates well with the degree of cognitive impairment [29]. Thus, [^18^F]FDG-PET and NOR test were performed for the in vivo evaluation of glucose metabolism and cognitive function in an AD transgenic mouse model. In vivo results showed that MSC-exosomes regulated the phase of neurons and astrocytes in the brain and rescued the deficits in glucose metabolism and cognitive function of aged AD transgenic mice. We also found that exosomal miR-29a could upregulate certain memory/synaptic plasticity-related genes via targeting the histone-modifying enzyme, histone deacetylase 4 (HDAC4), suggesting that HDAC4 might be a critical molecule for AD diagnosis and treatment.

## 2. Materials and Methods

### 2.1. Isolation and Characterization of MSC-Exosomes

Wharton’s jelly MSCs (WJ-MSCs, obtained by BCRC in Taiwan) were cultured in αMEM supplemented with 10% fetal bovine serum (Hyclone, Logan, UT, USA), 1% penicillin–streptomycin, and 1% L-Glutamine. Once WJ-MSCs reached 60–70% confluence, the medium were replaced with α-MEM containing exosome-depleted FBS. The exosome-depleted FBS was obtained by ultracentrifugation at 100,000× *g* at 4 °C overnight. Conditioned medium was collected on the second day (90% confluence) and filtered through a 0.22 μm filter (Millipore, Bedford, MA, USA) to sieve out dead cells and large growth debris. The filtered supernatant was concentrated by 100 kDa Amicon^®^ Ultra-15 Centrifugal Filter Units (Millipore, Bedford, MA, USA). Then, Exo-Prep kit (Lonza, Basel, Switzerland) was used to isolate exosomes, followed by purification with PD-10 columns (Sephadex G-25 in PD-10 Desalting Columns, GE Healthcare, Milwaukee, WI, USA). After that, the exosomes were eluted with PBS for further characterization and for use in experiments. The exosomes were filtered with a 0.22 μm filter and stored in a −80 °C freezer until use. The protein content of the exosome fraction was assessed by the Bradford method. The structure and size of WJ-exosomes were determined by transmission electron microscopy (TEM) and analyzed by nanoparticle tracking analysis (NTA) [30]. The presence of exosome-associated markers including CD9 (CBL162, Millipore, Bedford, MA, USA), CD63 (CBL553, Millipore, Bedford, MA, USA), and HSP70 (Cat. 386032, Sigma-Aldrich, St. Louis, MO, USA) in each batch of the exosome preparation was verified following the standards recommended by the International Society of Extracellular Vesicles (ISEV) [31]; calnexin (AB2301, Millipore, Bedford, MA, USA) served as a negative marker.

### 2.2. Labeling and Tracking of MSC-Exosomes

To track the trajectory of WJ-exosomes after injection into mice, the fresh harvested exosomes were labeled with a red fluorescent dye PKH26 (Sigma-Aldrich, St. Louis, MO, USA). Briefly, 1.5 mg of exosomes were incubated with a mixture of diluent c solution and PKH26 dye for 10 min, and then exosome samples were filtered through Amicon ultra-0.5 mL centrifugal filters (Millipore, Bedford, MA, USA). After washing with PBS twice, exosomes were resuspended in 500 μL PBS. These PKH26 dye-labeled exosomes were immediately injected intravenously, and the whole brain of AD mice was observed after 3 h by ex vivo imaging with an IVIS^®^ 50 imaging system (Perkin Elmer, Waltham, MA, USA).

### 2.3. Establishment of the FAD Human Neural Cell Culture Model

The human neuroblastoma cell line SH-SY5Y (a gift from Prof. Irene Han-Juo Cheng, National Yang Ming Chiao Tung University, Taiwan) was cultured in 1:1 Dulbecco’s modified Eagle’s medium and Nutrient Mixture F12 containing 10% FBS, 2 mM glutamine, 100 units/mL penicillin, and 100 μg/mL streptomycin at 37 °C in a 5% CO_2_-containing atmosphere. We overexpressed human APP with both K670N/M671L (Swedish) and V717I (London) FAD mutations using lentivirus in SH-SY5Y cells, which have been frequently used in AD studies, and grew these cells using a Matrigel culture model (see Supplementary Materials). Thin-layer (100–300 μm) and thick-layer (about 4 mm) cultures were set up, respectively, for subsequently IF and biochemical analyses. For neuronal differentiation, cells were pre-differentiated with 5 μM retinoic acid (RA, Sigma-Aldrich, St. Louis, MO, USA) for 1 week and then mixed 1:1 with pre-chilled Matrigel (Cat. 354234, Corning, Tewksbury, MA, USA) and dispensed into each tissue culture insert in 24-well plates at a density of 5 × 10^4^ cells/well for thick-layer culture [32]. Cells were maintained 2–8 weeks for differentiation after adding a combination of retinoic acid (5 μM) and BDNF (50 ng/mL). Moreover, mixtures of pre-differentiated cell/Matrigel (ratio 1:10) were seeded onto coverglass plates for IF and microscopy analysis.

### 2.4. Administration of MSC-Exosomes In Vitro and In Vivo

In vitro study: To examine the effect of WJ-exosomes, 50 μg of WJ- exosomes were applied twice a week to FAD-differentiated cell cultures. An equal amount of PBS employed for the dilution of exosomes was used as a control. Finally, total RNA was extracted, and IF staining was performed.

In vivo study: 9-month-old Tg mice and WT mice were divided into 2 groups: 50 μg of WJ-exosomes and PBS; 6 mice in each group. These mice were weekly intravenously injected with a dose of 50 μg of purified MSC-exosomes or PBS for 4 weeks. [^18^F]FDG PET imaging and behavior tests (NOR test) were performed before/after the injection of MSC exosomes. At the end, all mice were sacrificed, and subsequently brain slices and total RNA were prepared following the manual’s procedure.

### 2.5. Immunoblotting

Cell lysates were extracted with RIPA supplemented with a protease inhibitor cocktail, and equal amounts of protein (30 μg) samples were separated in SDS-PAGE gels. For immunoblotting, membranes were probed with primary antibodies for Aβ (Cat. 8243, Cell signaling, Danvers, MA, USA and clone 6E10, Covance Inc, Princeton, NJ, USA), NeuN (Cat. 52283, Arigo, Taiwan), Nestin (Cat. 52345, Arigo, Taiwan), Neprilysin (Ab5458, Millipore, Bedford, MA, USA), or GAPDH (Cat. 10112, Arigo, Taiwan) overnight at 4 °C and then incubated with horseradish peroxidase (HRP)-conjugate goat anti-mouse IgG (1:10000, ab136815, Abcam, Cambridge, UK) or HRP-conjugate goat anti-rabbit IgG (1:1000, ab6721, Abcam, Cambridge, UK). Protein signals were visualized using a chemiluminescent HRP substrate detection system (BioRad, Hercules, CA, USA) and acquired by a luminescence imaging system (UVP BioSpectrum 600, Thermo Fisher Scientific Inc., Waltham, MA, USA).

### 2.6. Reverse Transcription and Quantitative Real-Time Polymerase Chain Reaction (PCR)

Total RNA of FAD-differentiated cells, brain tissue, and MS- derived exosomes was extracted using TRIzol reagent (Invitrogen, Carlsbad, CA, USA). These RNA samples were used for reverse transcription and subsequent PCR amplification using the SuperScript III First-Strand Synthesis System (Thermo Fisher Scientific Inc., Waltham, MA, USA) and SYBR Green PCR Master Mix (Invitrogen, Carlsbad, CA, USA). The housekeeping gene β-actin was also amplified in parallel as a reference for the quantification of the transcripts. For the quantification of miRNA in WJ-MSC-derived exosomes, qRT-PCR was performed according to the instructions provided with the miScript PCR Starter kit (Qiagen, Hilden, Germany). Known miRNAs were used as miRNA-specific 5′ primers, and universal primers supplied with the kit were used as 3′ primers. The U6 primer was detected as a control. Quantitative real-time PCR was performed with the StepOne^TM^ Real-Time System PCR system (Thermo Fisher Scientific Inc., Waltham, MA, USA) according to the manufacturer’s recommendations. The specific primers are listed in Table S2. The average threshold cycle (Ct) for each gene was normalized to the Ct of β-actin or U6.

### 2.7. Animals and Genotyping

The J20 mouse model of AD (JAX-006293) and an age-matched control group were used in this study. The protocol was approved by the Committee on the Ethics of Animal Experiments of the National Yang Ming Chiao Tung University (IACUC number: 1070614, permission date: 28 June 2018). Genomic DNA was purified from tail biopsies by isopropanol precipitation, and the transgene was amplified by PCR using the forward primer GGTGAGTTTGTAAGTGATGCC and the reverse primer TCTTCTTCTTCCACCTCAGC. Resulting PCR products of 360 base pairs (bp) were analyzed by 1.5% agarose gel electrophoresis.

### 2.8. Small Animal PET/CT Imaging Experimental Procedures

In the PET/CT image study, each mouse was injected intravenously with 7.4 ± 1.2 MBq/0.1 mL of [^18^F]FDG for assessment of brain glucose metabolism or with 39.5 ± 3.5 MBq/0.2 mL of [^11^C]Pittsburgh compound-B (PiB) for detection of Aβ deposition. Static images were acquired after injection by the Triumph PET/SPECT/CT imaging scanner (Gamma Medica-Ideas Inc, Northridge, CA, USA). Anesthesia was induced and maintained using 1–1.5% isoflurane and 100% O_2_ inhalation through a nose cone, and all efforts were made to minimize suffering. A PET images dataset was then reconstructed using the ordered-subset expectation maximization algorithm with standard-mode parameters and the 2D maximum likelihood expectation maximization algorithm. Subsequently, regional retention and uptake of these radioligands were processed and analyzed with PMOD 3.5 software package (Pmod Technologies, Zurich, Switzerland). The values were reported as standardized uptake values (SUV) which represented the mean activity values for each whole brain or regional uptake, normalized to the injected dose per body weight of each individual animal.

### 2.9. Novel Object Recognition (NOR) Test

The NOR test is a memory and cognitive test. The task started with a habituation trial during which the animals were placed in an empty arena (40 × 40 cm, 30 cm high) for 5 min. The next day, mice were again placed in the same arena containing two identical objects (familiarization phase). Sniffing, touching, and stretching the head toward the object at a distance of no more than 2 cm were scored as object investigation. After 24 h (test phase), mice were again placed in the arena containing two objects, one of them already presented during the familiarization phase (familiar object) and a new different object (novel object), and the time spent exploring the two objects was recorded for 5 min. Results were expressed as percentage of time of investigation on objects per 5 min or as discrimination index (DI), i.e., (seconds spent on novel−seconds spent on familiar)/(total time spent on objects).

### 2.10. Dissection of Mouse Brain

Each mouse was perfused transcardially with cold saline (0.9%) followed by 4% paraformaldehyde. The fixed brain was immediately removed from the skull, and post-fixed by immersion in 4% paraformaldehyde for 3 days. The cerebral cortex of one hemisphere was dissected for total RNA or protein extraction, while the other hemisphere was cryoprotected for 3 days in a 30% sucrose solution and cut into 10 μm sagittal sections.

### 2.11. Immunofluorescence (IF) Staining

Briefly, after washing with PBS twice and fixing with 4% formaldehyde, brain sections or cell culture slides were incubated with the primary antibodies overnight at 4 °C, and then incubated with Alexa 594 or Cy5-conjugated secondary antibodies (Abcam, Cambridge, UK) and DAPI for 1 h in the dark room at room temperature followed by 10 min PBS washing for 3 times. All slides were mounted with ProLong Antifade Mounting Medium (catalog #P36970, Life Technologies, Carlsbad, CA, USA) and covered with cover slides before visualization under a confocal fluorescence microscopy. The following primary antibodies were used: monoclonal antibody against microtubule-associated protein 2 (MAP2, 1:500, Millipore, Bedford, MA, USA); NeuN (1:250; Cell signaling, Danvers, MA, USA); HDAC4 (1:400; Abcam, Cambridge, UK); monoclonal antibody against glial fibrillary acidic protein (GFAP, 1:250; Abcam, Cambridge, UK); polyclonal antibody against β-amyloid antibody (Aβ, 1:400; Cell signaling, Danvers, MA, USA). For detection of PKH26-labeled MSC-exosomes in the brain, the brain slices were imaged under a 40×, 63× and 100× objective after DAPI counterstaining using a confocal microscope with laser sources of 594 nm.

### 2.12. Image Acquisition and Quantification

Five images from random fields were obtained and analyzed using a laser-scanning confocal microscope (Zeiss LSM 880, Jena, Germany) with a CCD camera (AxioCam 503 MONO, Jena, Germany) using Zen Blue software (ZEISS, Jena, Germany). Plaque deposition was examined on Tg mice using the monoclonal anti-Aβ antibody as described above and was quantified using ImageJ (NIH). An intensity threshold level was set for stained plaques, and ROIs were measured for the areas covered by plaque staining. The total area of plaque loads was obtained from each section measured and divided by the total area of that region. The percentage of plaque load for each region was averaged from all mice in a group and compared between the different groups. For quantification of GFAP or HDAC4 immunostaining, all images were acquired within hippocampus or prefrontal cortex, which was selected randomly. Fluorescent intensities and cell numbers were quantified by Image Pro Plus ver4 (Media cybernetics, Rockville, MD, USA).

### 2.13. Statistical Analysis

Quantitative results were expressed as mean ± SEM. Data were subjected to one-way analysis of variance (ANOVA) followed by Student’s *t*-test, as appropriate, with GraphPad Prism v.5.0 software (GraphPad, San Diego, CA, USA). Quantitative PCR data were analyzed by Student’s *t*-test. Comparisons of different parameters between the groups were made by a post hoc analysis using a Tukey’s test. A P value less than 0.05 was considered statistically significant.

## 3. Results

### 3.1. Characterization of MSC-Derived Exosomes

**Figure 1:**
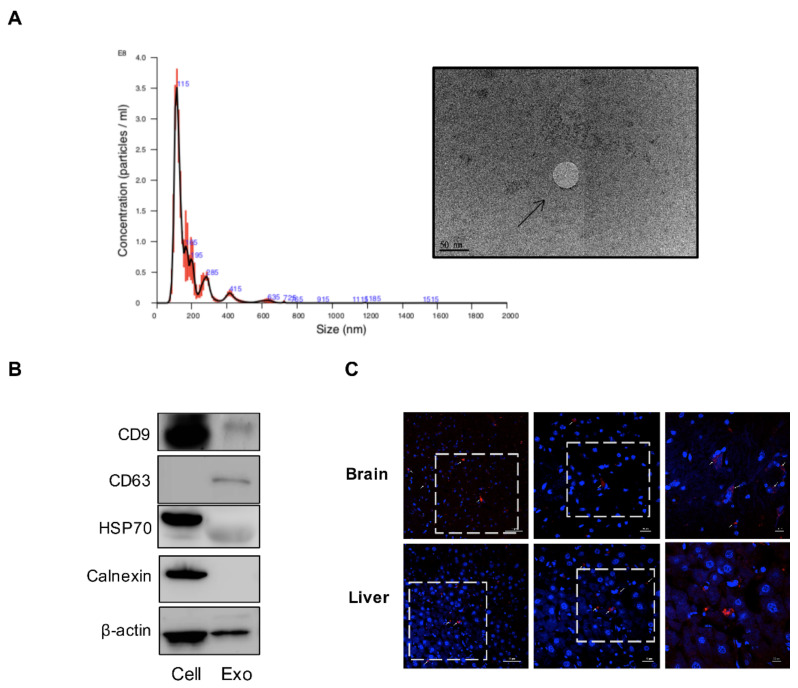
Characterization of MSC-exosomes. (**A**) TEM analysis of purified MSC-exosomes. The arrow indicates MSC-exosomes; scale bar: 50 nm. Size distribution of exosomes as measured by nanoparticle tracking analysis (NTA) showed a peak at 155 nm. (**B**) Western blot analysis of the proteins in MSC-exosomes (Exo) and MSCs (cell). (**C**) The slices were imaged under 40×, 63×, and 100× objectives after DAPI counterstaining, using a confocal microscope with laser sources of 594 nm. The white-dashed square represents a field-of-view corresponding to the image with higher magnification. Arrows indicate PKH26-labeled MSC-exosomes. Scale bars, 50 μm (top), 20 μm (middle), and 10 μm (bottom). Abbreviation: TEM, transmission electron microscopy; MSC, mesenchymal stem cell; HSP 70, heat shock protein 70.

To confirm the therapeutic potential of MSC-exosomes in AD, we isolated exosomes from Wharton’s jelly MSCs (WJ-MSCs), which are derived from the umbilical cord. MSC-exosomes showed a morphologically round shape as visualized by transmission electron microscopy (TEM). The size distribution was physically homogeneous with a peak at 115 nm (Figure 1A). Western blot analysis showed three positive protein markers of exosomes, including transmembrane/lipid-bound protein, CD9, and CD63, and one cytosolic protein, Hsp70. Additionally, calnexin was used as a negative marker of exosomes (Figure 1B). We next characterized the specific compositions of MSC-exosomes, such as proteins and miRNAs. Given that neurotoxic Aβs induces cell damage to a greater extent during long-term disease progression, strategies to target Aβs may alleviate AD pathology. Neprilysin (NEP), a membrane-anchored zinc metalloendopeptidase, can degrade Aβ monomers and Aβ oligomers. Interestingly, the expression and function of NEP are decreased significantly in AD patients [33]. We ensured that the MSC-exosomes expressed active NEP on their membranes, as shown by western blot and fluorometric NEP-specific activity assay (Figure S1A).

Numerous reports have described that exosomes can be used to treat various diseases through delivery of miRNA [34,35,36,37]. We used NGS to comprehensively examine the levels of miRNA expression; the top 20 most highly expressed miRNAs are listed in Table S1. We also validated the expression of three miRNAs by real-time RT-PCR (Figure S1B). Finally, to confirm whether intravenously injected MSC-exosomes crossed the BBB and entered the brain with AD, we tracked MSC-exosomes labeled with PKH26 fluorescent dye and assessed their biodistribution by ex vivo fluorescent imaging [38,39]. MSC-exosomes are considered to accumulate in the liver more efficiently; however, in our study, the fluorescent signals were also detected in the brain of AD mice (Figure S1C). Confocal microscopic analysis revealed that the MSC-exosomes identified by fluorescent particles were randomly distributed in the brain (Figure 1C). Taken together, these MSC-exosomes with thorough characterization were used to treat AD pathology in vitro and in vivo.

### 3.2. MSC-Exosomes Decrease the Level of Aβs and Prevent the Downregulation of Neuronal Memory/Synaptic Plasticity-Related Genes

Considering the pivotal role of Aβs, our study aimed to reduce the levels of elevated Aβ and thus ameliorate AD pathology. A human neural cell culture model consisting of a human neuroblastoma cell line overexpressing human APP with double FAD mutations was established in vitro (Figure S2). We first evidenced the obvious Aβ expression, Tau phosphorylation, and downregulation of learning, memory, and synaptic plasticity-related genes including brain-derived neurotrophic factor (*Bdnf* exon IV) and *Homer1*, that are involved in learning and memory formation, and of genes implicated in synaptic plasticity, such as those for the glutamate receptor subunits *GluR1*, *GluR2*, *NR2A*, and *NR2B* (also known as *Grin2a* and *Grin2b*), and *Syp* (synaptophysin) [40]. Significantly, lower expression of these genes, 53.7% (*BdnfIV*), 16.2% (*SYP*), 93.6% (*GluR2*), and 18.8% (*GRIN2B*), were observed in differentiated FAD cells compared to WT cells (Figure S2C).

**Figure 2:**
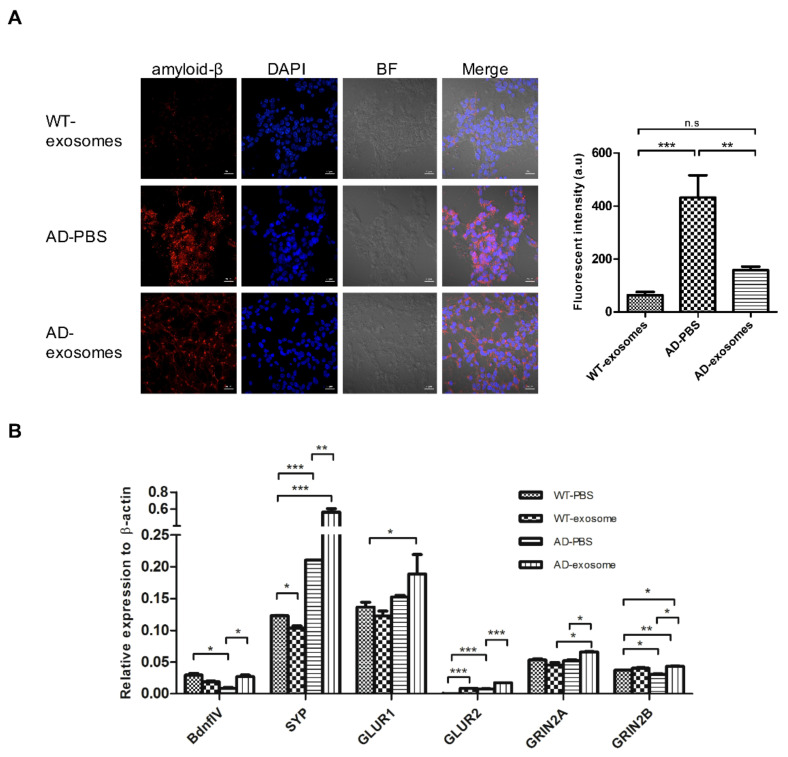
MSC-exosomes decrease the level of Aβ and prevent the downregulation of memory/synaptic plasticity-related genes. (**A**) Representative images of Aβ expression in the FAD cell culture model after one-week treatment and quantitative data of the average Aβ expression. Red: Aβ; Blue: DAPI; BF, bright field. Scale bar, 20 μm. Right graph depicts the quantification of five random areas in arbitrary units. Data are mean ± SEM. (* *p* < 0.05, ** *p* < 0.01, *** *p* < 0.001 by Tukey’s post hoc test, n.s., nonsignificant) (**B**) Quantitative RT-PCR results of plasticity-related genes. Data are expressed as mean ± SEM.

To further examine whether MSC-exosomes reduce Aβ expression and restore the normal level of these genes, differentiated FAD cells were incubated with or without MSC-exosomes for one week. We observed not only that the level of Aβs was reduced but also that the level of most plasticity-related genes was significantly higher in MSC-exosomes-treated FAD cells than in cells without treatment, and even comparable with levels in WT cells (Figure 2). One-way analysis of variance (ANOVA) revealed a significant effect of MSC-exosomes treatment (F = 11.2, *p* = 0.022. ** *p* < 0.01 by Tukey’s post hoc test). These data demonstrated that MSC-exosomes contribute to a decrease in Aβ expression, along with the recovery of neuronal memory and synaptic plasticity-related genes.

### 3.3. MSC-Exosomes Improve Brain Glucose Metabolism and Cognitive Function

**Figure 3:**
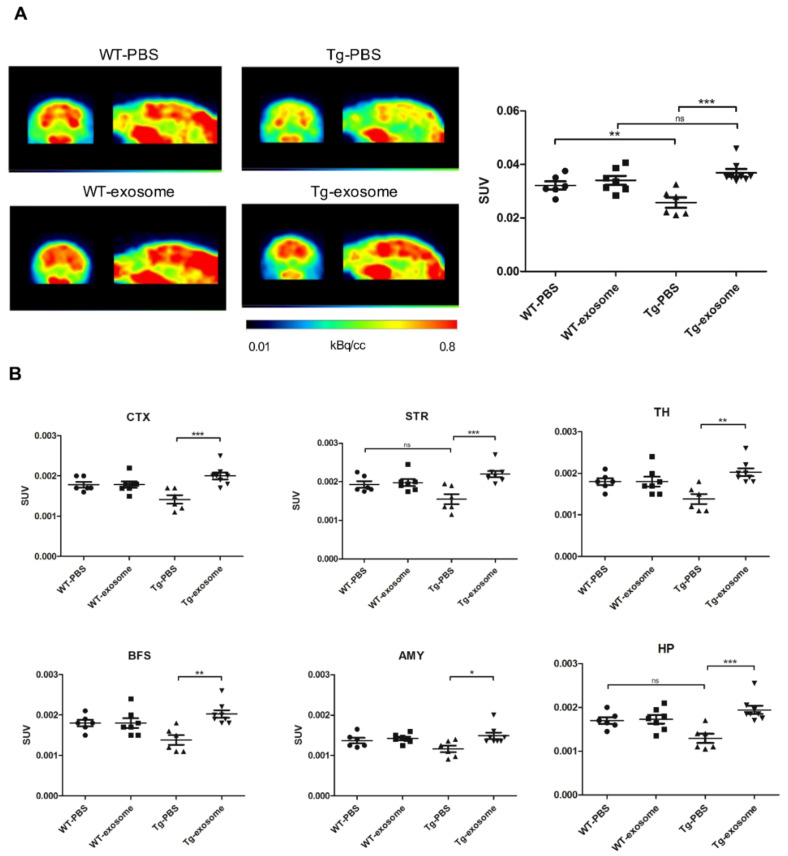
MSC-exosomes enhance [^18^F]FDG uptake in the whole brain and in different brain regions. (**A**) Representative [^18^F]FDG PET imaging of an AD transgenic mouse and a WT mouse (*n* = 6–8/each group). Sagittal (right) and coronal (left) slices were projected on a built-in T1 MRI mouse template (scale by kBq/c.c). Right graph: quantification of whole brain in SUV. Data are expressed as mean ± SEM; n.s., Nonsignificant. (* *p* < 0.05, ** *p* < 0.01, *** *p* < 0.001 by Tukey’s post hoc test) (**B**) [^18^F]FDG uptake in different brain regions in Tg and wild-type (WT) mice. SUV means standardized uptake value. Abbreviation: CTX, cortex; TH, thalamus; BFS, basal forebrain; STR, striatum; HP, hippocampus; AMY, amygdala.

J20 AD transgenic (Tg) mice, harboring Swedish (K670N/M671L) and the Indiana (V717F) mutations of the amyloid precursor protein, exhibit an age-related and progressive neuropathological phenotype that presents both plaque and tangle pathology [41]. According to our longitudinal study, an obvious Aβ deposition (detected by [^11^C]PiB PET imaging) and a tendency to decreased glucose metabolism were manifested at nine months of age (Figure S3). We next investigated the therapeutic effects of MSC-exosomes on AD-type pathologies and cognitive deficits in nine-month-old J20 AD Tg mice. [^18^F]FDG-PET and NOR test were performed to evaluate glucose metabolism and cognitive function, respectively, before/after treatment with MSC-exosomes. Visual interpretation of [^18^F]FDG-PET images indicated a recovered signal throughout different areas of the brain (sagittal and coronal slices), and quantitative analyses confirmed a significant increase (+43%; *p* = 0.0012) in the global cerebral FDG-PET signal in MSC-exosomes-treated Tg mice compared to mice treated with PBS controls (Figure 3A). Whole brains were also anatomically parcellated according to a standard atlas, and [^18^F]FDG uptake in each region of amygdala, basal forebrain, hippocampus, cortex, thalamus, and striatum was evaluated (Figure 3B). Tg mice without MSC-exosomes exhibited significantly lower [^18^F]FDG uptake in all areas examined, including amygdala (*t* = 2.83, *p* = 0.0164), basal forebrain (*t* = 4.22, *p* = 0.0018), hippocampus (*t* = 4.65, *p* = 0.0007), cortex (*t* = 4.34, *p* = 0.0015), thalamus (*t* = 4.22, *p* = 0.0018), and striatum (*t* = 4.21, *p* = 0.003). There was no significant difference in [^18^F]FDG uptake between MSC-exosomes-treated WT mice and PBS-treated WT mice in any of the regions examined.

**Figure 4:**
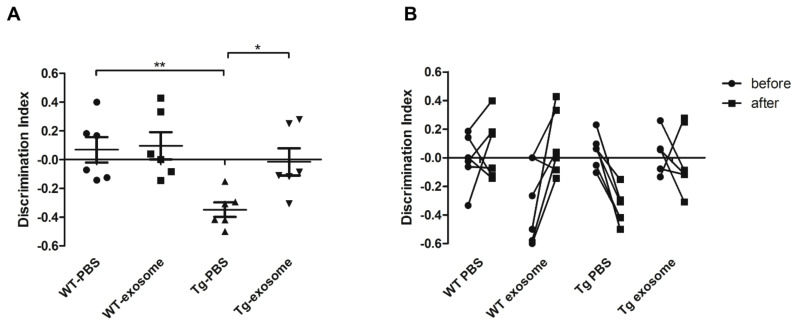
MSC-exosomes improve cognitive function. (**A**) After MSC-exosomes treatment, the NOR test was used to assess the long-term recognition memory in all groups (10 months of age). Histograms are mean ± SEM of the corresponding DI (Discrimination index). One-way ANOVA revealed a significant effect of treatment (F = 5.93, *p* = 0.0046. * *p* < 0.05, ** *p* < 0.01 by Tukey’s post hoc test). (**B**) The NOR test was performed before and after MSC-exosomes treatment. Two-way ANOVA was used, and a significant interaction was found between the factors Tg × treatment (*p* = 0.0049 by Bonferroni post hoc test).

The effect of MSC-exosomes on cognitive function was further evaluated. Compared with PBS-treated Tg mice, MSC-exosomes-treated Tg mice performed better in the NOR test, as reflected by a significant increase in the discrimination index (DI). In addition, MSC-exosomes treatment promoted recovering of long-term recognition memory in Tg mice (percentage of time of investigation per 5 min: familiar, 15.5 ± 3.4; novel, 14 ± 5.4; DI, −0.083 ± 0.3.16), to values close to those recorded for PBS-treated WT mice (percentage of time of investigation per 5 min: familiar, 18.2 ± 12.22; novel, 20.5 ± 10.78; DI, −0.0157 ± 0.23) (Figure 4A). One-way ANOVA for the DI showed a significant effect of treatment (F = 5.93, *p* = 0.0046). We also observed that the MSC-exosomes treatment had no negative effect on the memory of WT mice (familiar, 12 ± 7.74; novel, 8.8 ± 3.83; DI, 0.131 ± 0.24). As shown in Figure 4B, we found that MSC-exosomes-treated Tg mice exhibited an increased in DI until 10 months of age, suggesting that the MSC-exosomes treatment rescued the cognitive decline. Taken together, our data suggest that MSC-exosomes were able to restore whole-brain regional glucose metabolism and improve cognitive function.

### 3.4. MSC-Exosomes Reduce Aβ Plaque Burden, Inhibit Astrocyte Activation, Upregulate Neuronal Memory and Synapse-Related Genes

**Figure 5:**
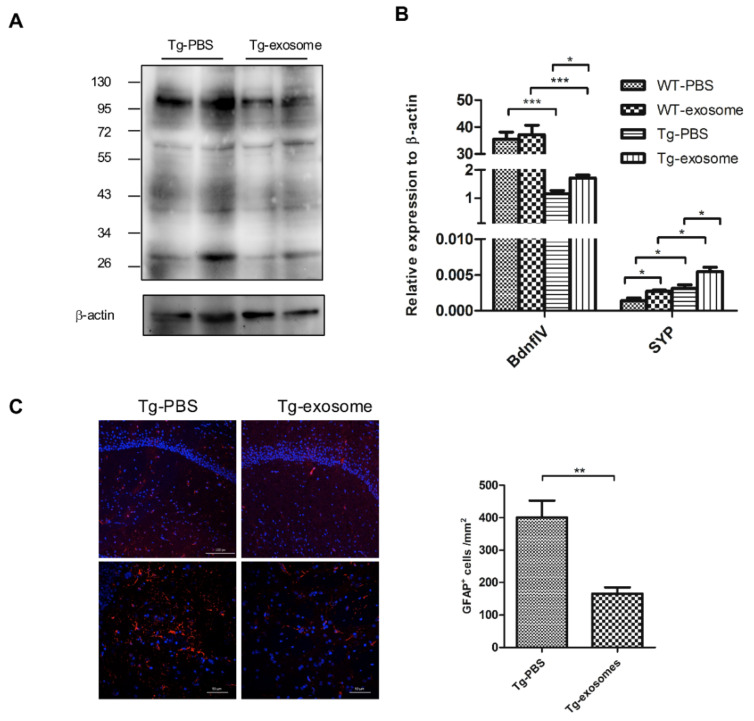
MSC-exosomes reduce Aβ plaque burden, inhibit astrocyte activation, and upregulate memory- and synapse-related genes. (**A**) Transgenic or WT mice were treated with MSC-exosomes or PBS for 4 weeks, and at the end of treatment, homogenized brain tissues were analyzed by SDS-PAGE and immunoblotting. Representative Western blot of soluble Aβ immunoprobed using Aβ antibody and visualized by ECL is shown. Internal control: β-actin. (**B**) Quantitative RT-PCR results of plasticity-related genes from extracted hemispheres. Data are mean ± SEM (* *p* < 0.05 and *** *p* < 0.001 by Student’s *t*-test). (**C**) Representative confocal micrographs of sagittal brain sections, immunolabeled with GFAP, in the hippocampus. Red, GFAP; Blue: DAPI. Scale bar, 100 μm (top), 50 μm (bottom). Quantification of GFAP^+^ cells within a region of interest (Area = 0.05 mm^2^). Data are mean ± SEM (*p* = 0.0043. ** *p* < 0.01 by Student’s *t*-test).

To assess whether MSC-exosomes decrease brain Aβ burden, brain homogenates, and sagittal slices were prepared. Western blot analysis showed that the treatment strongly reduced the levels of soluble Aβ species with MW up to 90 kDa, which are generally considered neurotoxic oligomers (Figure 5A; −38.2%, *p* < 0.001). To examine the effect of MSC-exosomes on the cellular phase of AD, the reactive astrocyte marker glial fibrillary acidic protein (GFAP), the neural maker NeuN, and the levels of neuronal memory/synaptic plasticity-related genes were analyzed. While AD transgenic mice without MSC-exosomes displayed an increase in reactive astrocytes until 10 months of age, the expression of GFAP decreased significantly in MSC-exosomes-treated Tg mice (Figure 5C and Figure S5A). No significant increase in NeuN expression was observed in the cortex or hippocampus (Figure S4A); nevertheless, quantitative real-time RT-PCR analysis of the extracted hemisphere showed increased levels of *BdnfIV*, *Syp*, and *GluR1* in MSC-exosomes-treated Tg mice compared to non-treated animals (Figure 5B and Figure S4B). Collectively, these results suggest that MSC-exosomes are beneficial on the cellular phase of the AD brain, which is in line with the improvement in cognitive functions.

### 3.5. MSC-Exosomes Downregulate HDAC4 Expression and Restore Target Genes

Generally, exosomes are deemed to have the ability to alter the molecular profile of recipient cells by transferring proteins, lipids, and genomic materials including mRNAs and miRNAs. MiR-29a, which specifically targets HDAC4, was found to be the most abundant miRNA in MSC-exosomes isolated from Wharton’s jelly MSCs. HDAC4, a histone-modifying enzyme, is highly expressed in the brain, and its homeostasis is linked to the transcriptional regulation of synaptic plasticity-related genes, neuronal survival, and neuron development. The nuclear HDAC4 level is markedly increased in the brains of AD patients, and the total HDAC4 level (cytoplasm and nuclear) is high in the AD mouse model [42,43], suggesting that abnormal HDAC4 expression or its nuclear localization may contribute to learning and memory deficits. Consistently, we observed a dose-dependent increase in HDAC4 expression in response to Aβ oligomers (Figure S5A). Within the FAD neural cell culture model, HDAC4 levels increased directly in proportion to Aβ levels (Figure S5B; r = 0.791, *p* = 0.0013).

**Figure 6:**
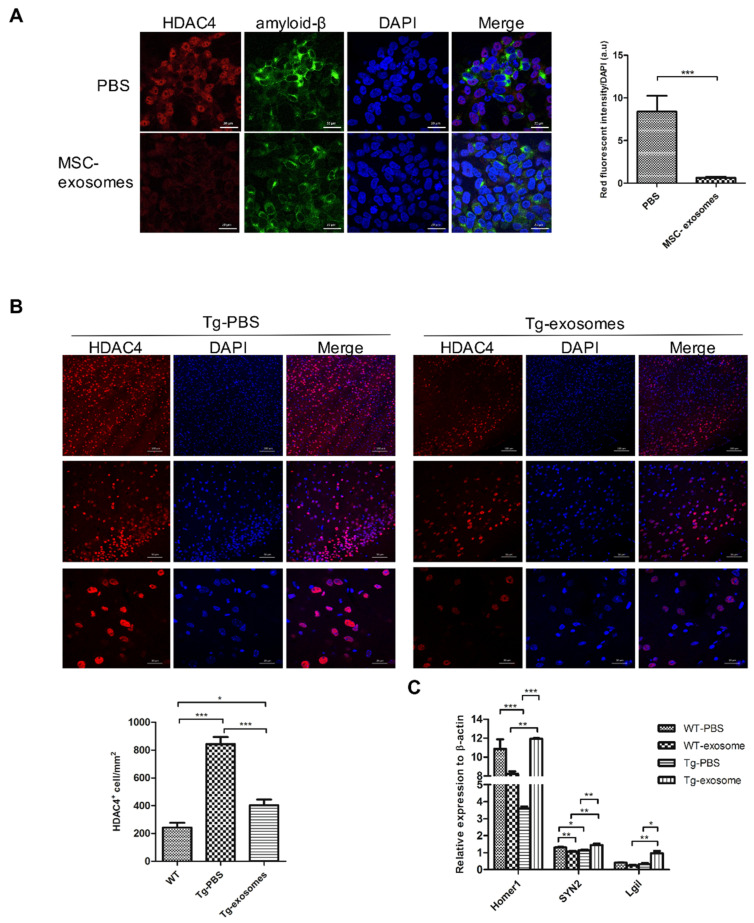
MSC-exosomes downregulate HDAC4 expression and restore the levels of target genes. (**A**) Representative confocal micrographs of a FAD cell culture immunolabeled with HDAC4. In total, 50 μg of MSC-exosomes was administered to differentiated cells twice a week. Red, HDAC4; Green: amyloid-β; Blue: DAPI. Quantification of HDAC4 labeling intensity in FAD-differentiated cells. Data are mean ± SEM (*** *p* < 0.001; n.s., nonsignificant by Student’s *t*-test). (**B**) Representative confocal micrographs of sagittal brain sections immunolabeled with HDAC4 in the cortex. Red, HDAC4; Blue: DAPI. Quantification of HDAC4^+^ cells within a region of interest (Area = 0.05 mm^2^). Data are mean ± SEM. The slices were imaged under 20×, 40× and 100× objectives after DAPI staining. Scale bars, 100 μm (top), 50 μm (middle) and 20 μm (bottom). (**C**) Quantitative RT-PCR results for HDAC4 target genes. Data are expressed as mean ± SEM. * *p* < 0.05, ** *p* < 0.01, *** *p* < 0.001 and n.s., nonsignificant by Student’s *t*-test.

To further assess the effect of exosomal miRNAs on HDAC4, we administered 50 μg of MSC-exosomes twice a week to differentiated cells. Compared to the control group (PBS), MSC-exosomes contributed to an obvious decrease in the level of Aβ and HDAC4 (Figure 6A, *p* < 0.0001). After administration of MSC-exosomes to Tg mice, the levels of nuclear HDAC4 decreased markedly (Figure 6B), and the expression of the HDAC4 target genes *Homer1* (Homer protein homolog 1), *Syn2* (Synapsin II), and *Lgi1* (leucine-rich glioma inactivated 1) were elevated compared to those in the WT group (Figure 6C). Collectively, these data suggest the potential of HDAC4 as a promising target for AD therapy.

## 4. Discussion

In this study, we demonstrated that exosomes isolated from Wharton’s jelly MSCs (MSC-exosomes) can act as a cell-free therapy for AD. In addition to the characterization of MSC-exosomes, we used in vitro and in vivo assays; a human neural cell culture model consisting of a human neuroblastoma cell line overexpressing human APP with double FAD mutations and the J20 AD transgenic mouse model were employed for the evaluation of the abovementioned MSC-exosomes therapy. Our study uncovered the considerable therapeutic benefits of MSC-exosomes, including (1) degradation of Aβ plaques, (2) improvement in brain glucose metabolism/cognitive function, and (3) modulation of epigenetics and genetic expression.

Aβ accumulation has been also hypothesized to be a result of an imbalance between Aβ production and clearance. Physiological metabolites of Aβ are constantly produced and removed in the brain, but Aβ clearance seems to be impaired, leading to Aβ deposition. Extracellular Aβ deposits can be removed from the brain by various clearance systems, such as blood–brain barrier (BBB) clearance, degradation clearance, and interstitial fluid bulk flow clearance [44]. In the degradation clearance system, extracellular Aβs can be degraded by proteases, such as neprilysin (NEP), matrix metalloproteinases (MMPs), and glutamate carboxypeptidase II. Thus, lowering brain Aβ levels by NEP may be a promising approach. We characterized Wharton’s jelly MSCs-derived exosomes by means of physical (NTA and TEM), biological (cell trafficking), and biochemical (western blot, NGS, and enzymatic ability) assays. The MSC-exosomes were shown to express NEP-specific enzymatic activity on their membranes. Our study also demonstrated that co-culturing of FAD neural cells with MSC-exosomes reduced the level of Aβ and prevented the downregulation of memory/synaptic plasticity-related genes. This therapeutic effect was satisfactory, but whether the reduction is directly mediated by NEPs needs to be further clarified.

[^18^F]FDG-PET imaging and cognitive assessment indicated a significant improvement in brain glucose metabolism and cognitive functions. In agreement with these results, memory/synaptic plasticity-related genes were upregulated after treatment. However, the number of Aβ plaques in MSC-exosomes-treated AD transgenic mice showed no significant change, unlike what observed in the FAD neural cell culture model. There are several reasons for the less remarkable degradation of Aβ plaques observed in the in vivo study. First, nine-month-old J20 AD Tg mice exhibited increased Aβ deposition to levels indicating clinical challenge (Figure S3A,B). However, in the described experiments, the treatment might have been administered too late to be able to degrade the dense-core plaques. It is worthwhile to perform a longitudinal study. Second, the dose of MSC-exosomes after intravenous administration was not high enough to degrade the plaques.

Despite our biodistribution study and others showed that MSC-exosomes have the ability to enter the brain of AD mice through intravenous administration, the amount of MSC-exosomes in the brain is limited compared to that in the liver (Figure 1C and Figure S1C) [25]. Several approaches have been investigated to improve the brain targeting ability of MSC-exosomes, including the utilization of central nervous system (CNS)-specific rabies viral glycoprotein (RVG) peptide-tagged exosomes, intranasal administration, and inducing permeability by a temporary disruption of the BBB [45,46,47]. Considering these modalities, we will optimize the therapeutic strategy and explore its exact mechanism in the future.

Considering the critical role of neurons in the CNS, dysfunctions in the brain with AD are mediated by a reduction in synaptic plasticity, changes in homeostatic scaling, and disruption of neuronal connectivity. Since brain uptake of FDG depends on the glucose transporters GLUT1 (highly expressed in astrocytes and endothelium of BBB), GLUT3 (neuron-specific), and glutamate transporter 1 (GLT-1), which also acts as one of the triggers for glucose uptake by astrocytes, [^18^F]FDG-PET signals reflect synaptic activity that is modulated by more than one cell type [48,49,50]. Astrocytes, which are involved in the development, maintenance, and repair of the neural environment, also contribute to cellular and functional degeneration in AD. Increased expression of GFAP and decreased level of GLT-1 and GLUT1 are the signatures of reactive astrocytes in the brain of AD patients as well as in AD mouse models [48,51,52]. In agreement with the improvement of glucose metabolism revealed by [^18^F]FDG-PET, reactive astrocytes were found to be inhibited, and neuronal memory/synaptic plasticity-related genes were upregulated post-treatment (Figure 5B,C). In our study, whole-brain analysis by IF staining and western blotting showed a slight increase in the expression of NeuN, which did not achieve statistical significance, after MSC-exosomes treatment (Figure S4A). Whether the MSC-exosome treatment can also regulate the neuronal growth or maturation observed in the current study needs to be further examined. Magnetic resonance imaging (MRI) is a more powerful diagnostic tool to create a brain connectivity map for the evaluation of the integrity of neuronal signal transduction [53]. A limitation of our study is the lack of multimodal imaging to evaluate the therapeutic effect of MSC-exosomes. Ideally, all parameters, including [^18^F]FDG-PET, cognitive test, and MRI, should have been measured in the same mouse at the same time point. However, this is technically highly challenging. Additionally, in AD, in response to toxic materials, reactive astrocyte (known as astrogliosis) exhibited morphological hypertrophy, proliferation, process ramification, and augmented expression of intermediate filaments (GFAP, vimentin, and nestin) [54]. In fact, astrogliosis is a relatively early event seen before Aβ deposition [51]. Increased monoamine oxidase B (MaoB) activity and GABA and Gs-protein-coupled adenosine receptor A_2A_ expression in astrogliosis are linked to memory defects. PET imaging with various radiotracers can be used to reveal astrogliosis in detail [55,56].

Studies have indicated that MSC-exosomes can induce anti-inflammatory effects by the inhibition of reactive astrocytes and activated microglia, the conversion of Th2 and Treg cells, as well as the release of cytokines [57]. Microglia, consisting of resident innate immune cells of the CNS, is one of the major players of neuroinflammation in AD. Recent microglial transcriptomes studies have shown that microglia subtypes in neurodegenerative disease, neuroinflammation, or aging can be identified in comparison with known signatures of homeostatic microglia, such as disease-associated-microglia (DAM), activated response-microglia (ARM), and interferon-response-microglia (IRM) [58]. Of note, there are widespread gene expression differences in different AD transgenic mouse models. Since we used J20 AD transgenic mice harboring APP mutations to evaluate the therapeutic effect of MSC-exosomes, the upregulated activated microglia marker genes in J20 mice are suitable for the investigation of the microglial response [59]. *Cst7* has been shown to be a marker for DAM and to be significantly upregulated along with AD progression. *Fcer1g*, encoding the Fc fragment of the IgE receptor, is significantly more highly expressed in ARM [60]. IRM is defined by the specific expression of *Ifit2* and *Ifitm3* [61]. As shown in Figure S6, the expression of these activated microglia marker genes was significantly upregulated in AD transgenic mice (J20) compared to wild-type mice. After MSC-exosomes treatment, the levels of *Cst7*, *Fcer1g*, *Ifit2*, and *Iftim3* were significantly decreased compared to the levels in non-treated mice (PBS). The results indicate that MSC-exosomes have the potential to inhibit activated microglia in AD, emphasizing their immunoregulatory function. Besides, whether oligodendrocytes and vascular system are regulated by MSC-exosomes needs further investigation.

MicroRNAs are endogenously expressed single-stranded RNAs of 20–22 nucleotides and repress their target gene expression by binding to complementary sequences within the 3′-untranslated region of the target mRNAs. In the past decade, synthetic miRNA mimics that imitate the function of endogenous miRNAs delivered by adenovirus-associated virus were used for cancer treatment [62]. However, the safety concerns of viral delivery systems limit their clinical applications. In recent years, the use of non-viral delivery systems, such as liposomes, polymeric nanoparticles, or conjugates of receptor-binding molecules has increased in gene therapy.

Exosomes are released by a variety of cells when multivesicular endosomes fuse with the plasma membrane and further serve as intercellular mediators [20,27,63]. Therefore, exosomes may have the potential to deliver miRNAs or other components to treat AD. According to the NGS analysis, miRNAs are enriched in MSC-exosomes isolated from Wharton’s jelly MSCs, especially HDAC4-targeting miRNA-29a. Particularly, we found an obvious decrease in the levels of nuclear HDAC4 and an increase in the expression of HDAC4-related genes after MSC-exosomes treatment. To confirm the regulatory effect of miRNA-29a on HDAC4, it is important to use miRNA-29a-deficient or -overexpressing MSC-exosomes for further studies.

The pathophysiological processes in AD are associated with the modulation of epigenetic mechanisms, such as DNA and histone modifications. The dynamic modification of histone acetylation is associated with synaptic plasticity and memory formation, and the importance of histone deacetylase (HDAC) enzymes in various cognitive processes has been emphasized [64]. Notably, global HDAC inhibitor (HDACi) treatments significantly restored impaired cognitive functions in mice [65]. This suggests that HDACs can serve as potential targets for AD therapy. To date, 18 HDACs have been identified in humans and are typically classified into four major classes [66]. Class I HDACs (e.g., HDACs 1, 2, 3, and 8) are small nuclear proteins usually involved in the regulation of cellular proliferation. According to protein structure and motif organization, class II HDACs are further categorized into two subclasses, class IIa with HDAC4, 5, 7, and 9, and class IIb with HDAC6 and 10. HDAC4, which is enriched in the brain, participates in regulating the transcription of synaptic plasticity-related genes, neuronal survival, and neuron development. However, the role of HDAC4 in AD is still unidentified. In line with previous studies, we found that nuclear HDAC4 is highly expressed in the AD mouse model, and its expression increases with age. Our study is the first to demonstrate that HDAC4 might serve as a critical molecule for a more complete depiction of AD progression.

## 5. Conclusions

This study helps to understand the potential therapeutic mechanisms of MSC-exosomes and provides a therapeutic platform based on cell-free exosomes to treat Alzheimer’s disease. Herein, we demonstrated that exosomes derived from Wharton’s jelly MSCs improved AD pathology, downregulated HDAC4, and regulated cellular phases simultaneously. We suggest that MSC-exosomes transfer biological materials, in particular, miRNAs, to regulate cellular and molecular mechanisms in the brain affected by AD. These results also highlight the beneficial effects of MSC-exosomes in AD treatment by tackling multiple key pathways of the disease pathogenesis. Our study suggests that MSC-derived exosomes have the potential to serve as a cell-free therapy for AD.

## Data Availability

The data presented in this study are available in article and supplementary material.

## Supplementary Materials

The following are available online at https://www.mdpi.com/article/10.3390/biomedicines9060594/s1, Figure S1: Characterization of MSC-derived exosomes-Cont’d, Figure S2: Establishment of a FAD human neural cell culture model as an in vitro platform, Figure S3: Longitudinal follow-up of Aβ deposition and metabolic function using [^11^C]PiB and [^18^F]FDG PET imaging in J20 transgenic mice, Figure S4: Effect of MSC-exosomes on neuron and plasticity-related genes in Tg mice, Figure S5: Aβ oligomers induce HDAC4, Figure S6: Effect of MSC-exosomes on activated microglial marker genes in Tg mice, Table S1: Top 20 most highly expressed miRNAs in MSC-exosomes according to NGS miRNA expression, Table S2: List of specific primers for memory/synaptic plasticity-related genes used in qPCR, Table S3: List of specific primers for activated microglia marker genes used in qPCR.
