# Supplementary material for: Mesenchymal Stem Cell-Derived Exosomes Ameliorate Alzheimer’s Disease Pathology and Improve Cognitive Deficits
Source: Biomedicines. 2021 May 24;9(6):594. doi: 10.3390/biomedicines9060594 (PMC8225157; doi:10.3390/biomedicines9060594)
Supplement: Supplementary file 1 [file biomedicines-09-00594-s001.zip › biomedicines-1205579-supplementary.pdf]

Mesenchymal Stem Cell Derived Exosomes Ameliorate Alzheimer's Disease Pathology and Improve Cognitive Deficits

Yi-An Chen[1, 2], Cheng-Hsiu Lu[2, 3], Chien-Chih Ke[2, 4, 5, 6, *], Sain-Jhih Chiu[2], Fong-Shya Jeng[2], Chi-Wei Chang[7], Bang-Hung Yang[7, 8], and Ren-Shyan Liu[1, 2, 7, 8, 9, *]

1. Institute of Clinical Medicine, National Yang Ming Chiao Tung University, Taipei 112, Taiwan

2. Molecular and Genetic Imaging Core/Taiwan Mouse Clinic, National Comprehensive Mouse Phenotyping and Drug Testing Center, Taipei 112, Taiwan

3. Industrial Ph.D Program of Biomedical Science and Engineering, National Yang Ming Chiao Tung University, Taipei 112, Taiwan

4. Department of Medical Imaging and Radiological Sciences, Kaohsiung Medical University, Kaohsiung 807, Taiwan

5. Drug Development and Value Creation Research Center, Kaohsiung Medical University, Kaohsiung 807, Taiwan

6. Department of Medical Research, Kaohsiung Medical University Hospital, Kaohsiung 807, Taiwan

7. National PET and Cyclotron Center (NPCC), Department of Nuclear Medicine, Taipei Veterans General Hospital, Taipei 112, Taiwan

8. Department of Biomedical Imaging and Radiological Sciences, National Yang Ming Chiao Tung University, Taipei 112, Taiwan

9. Department of Nuclear Medicine, Cheng Hsin General Hospital, Taipei 112, Taiwan

* Correspondence: Ren-Shyan Liu, National Yang Ming Chiao Tung University, Taipei 112, Taiwan, ROC. E-mail: rsliuvgh@gmail.com. Tel: 886-2-28757301 ext. 575. Fax: 886-2-28749431; Chien-Chih Ke, Department of Medical Imaging and Radiological Sciences, Kaohsiung Medical University, Kaohsiung, Taiwan, ROC. E-mail: s2289.tw@yahoo.com.tw

Supplementary Information Index

Figure S1) Characterization of MSC-derived exosomes-con't

Figure S2) Establishment of a FAD human neural cell culture model as an *in vitro* platform

Figure S3) Longitudinal follow-up of Aβ deposition and metabolic function using $[^{11}C]$PiB and $[^{18}F]$FDG PET imaging in J20 transgenic mice

Figure S4) Effect of MSC-exosomes on neuron and AD related plasticity-related genes in Tg mice

Figure S5) Aβ oligomers induce HDAC4

Figure S6) The effect of MSC-exosomes on activated microglial marker genes in Tg mice

Table S1) Top 20 most highly expressed miRNAs in MSC exosomes according to NGS miRNA expression

Table S2) List of specific primers for memory/synaptic plasticity-related genes used in qPCR

Table S3) List of specific primers for activated microglia marker genes used in qPCR

Materials and methods

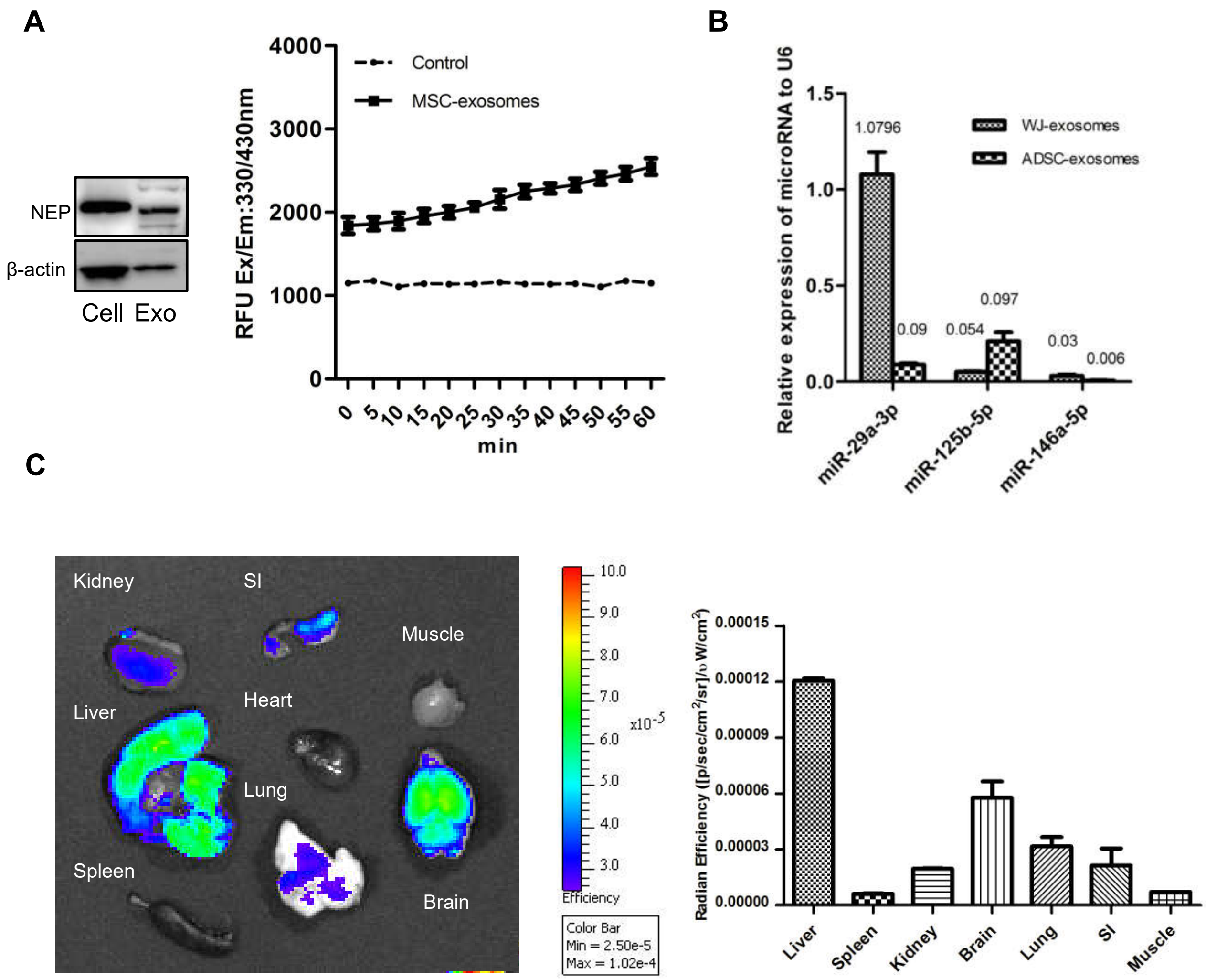

Figure S1. Characterization of MSC-derived exosomes- Cont'd. (A) Neprilysin expression of MSC-derived exosomes. Left panel: Western blot analysis of NEP expression in MSC-exosomes (Exo) and MSCs (cell). Right panel: NEP-specific enzyme activity was measured in MSC-exosomes. The average NEP activity represented by fluorescence intensity was 93.8±13.2 μU/mg measured with a reading interval of 5 min. Control: PBS; Data are expressed as mean±SD. (B) Detection of miRNAs in MSC exosomes using real time PCR. Quantification analysis of miRNAs in WJ-MSC exosomes and ADSC derived exosomes. Transcript levels were normalized to U6 levels. Data are the mean ± S.D. ADSC, adipose derived stem cell (C) Left panel: Brain of AD mouse was harvested for *ex vivo* fluorescent imaging with IVIS 50 at 3 hrs post injection. Right panel: Quantification of fluorescent signals in each organ.

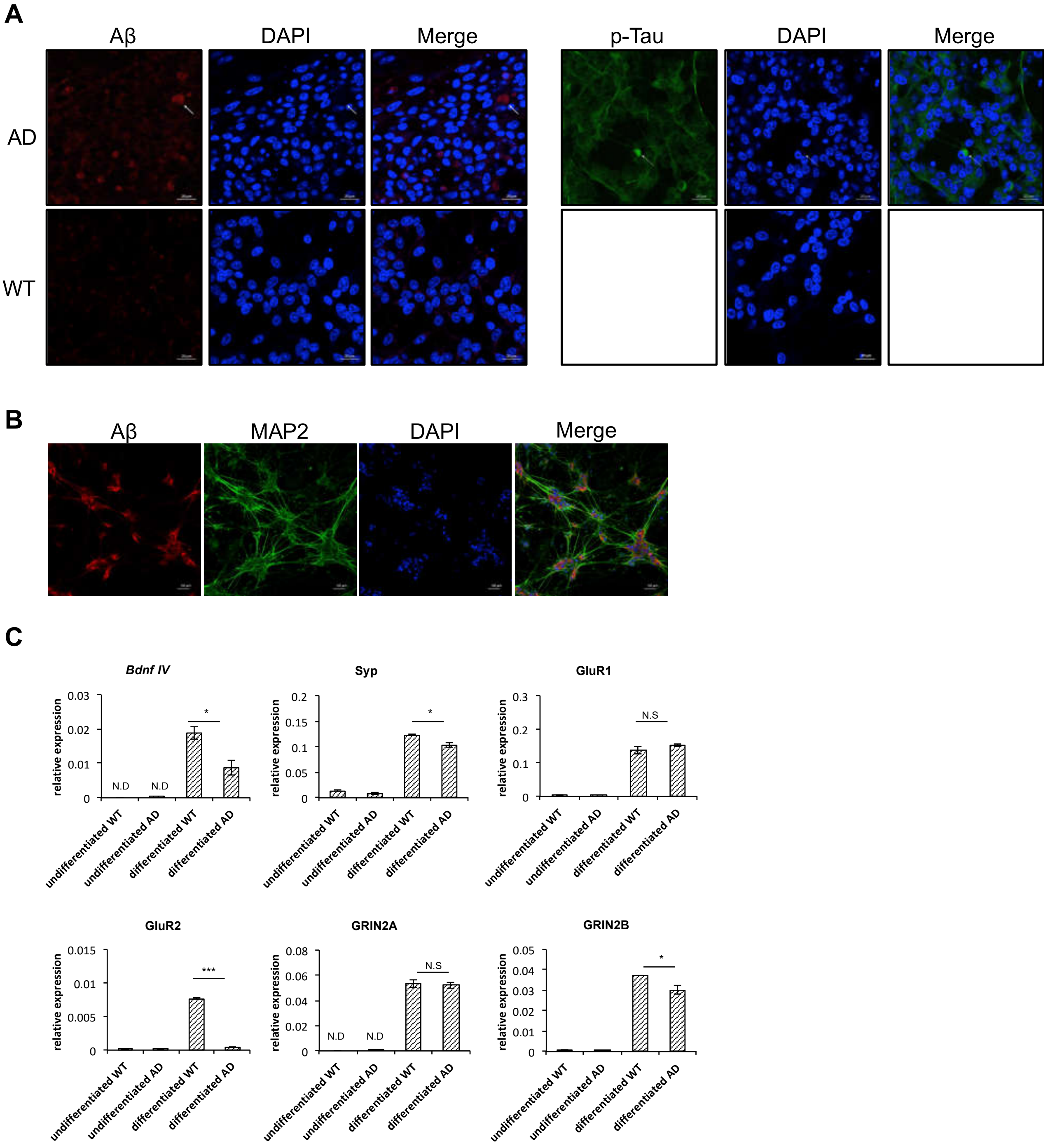

Figure S2. Establishment of a FAD human neural cell culture model as an *in vitro* platform. (A) IF staining against β-amyloid and p-tau (S396 and S404) in 7-week differentiation. Blue, DAPI; scale bar, 20 μm. (B) Detection of mature neuron marker, MAP2 and amyloid-β. Scale bar, 50 μm. (C) Quantitative RT-PCR results of AD related affected genes. Data are expressed as mean ± SEM (*$p < 0.05$, **$p < 0.01$, ***$p < 0.005$ by Student's t test). N.D, no detected; N.S, nonsignificant.

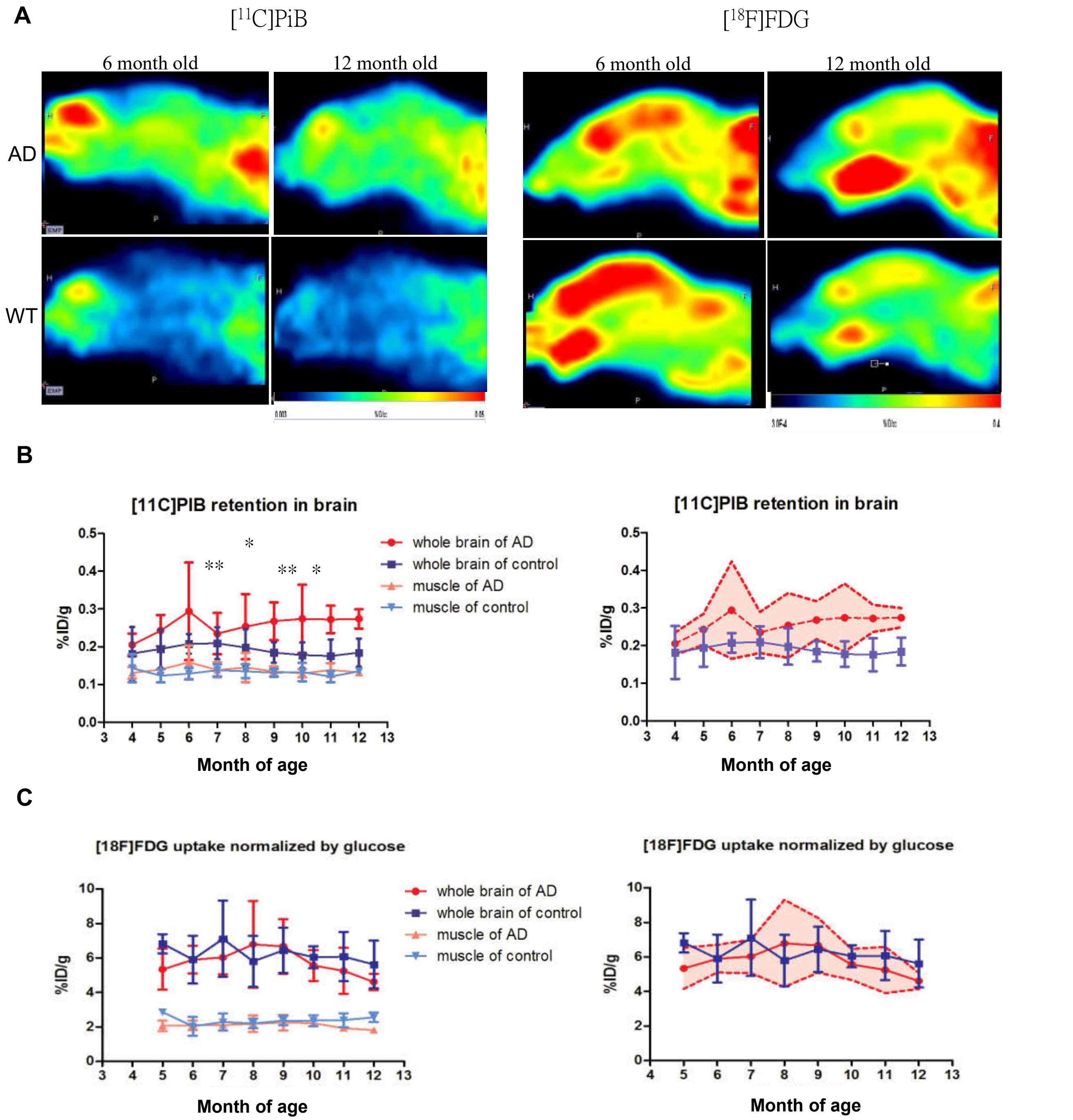

Figure S3. Longitudinal follow-up of Aβ deposition and metabolic function using [^{11}C]PiB and [^{18}F]FDG PET imaging in J20 transgenic mice. (A) Representative [^{11}C]PiB and [^{18}F]FDG PET imaging of AD transgenic mouse and WT mouse (n=6/each group) at 6 and 12 month of age respectively. Sagittal slices were projected on T1 MRI mouse template (scale by %ID/c.c). (B) Aβ deposition was detected by [^{11}C]PiB monthly starting at 4 month of age. PET images based quantitative analysis of

[11C]PiB retention in whole brain (n=6). Control group: age-matched WT mice. (C) PET images based quantitative analysis of [18F]FDG uptake in whole brain.

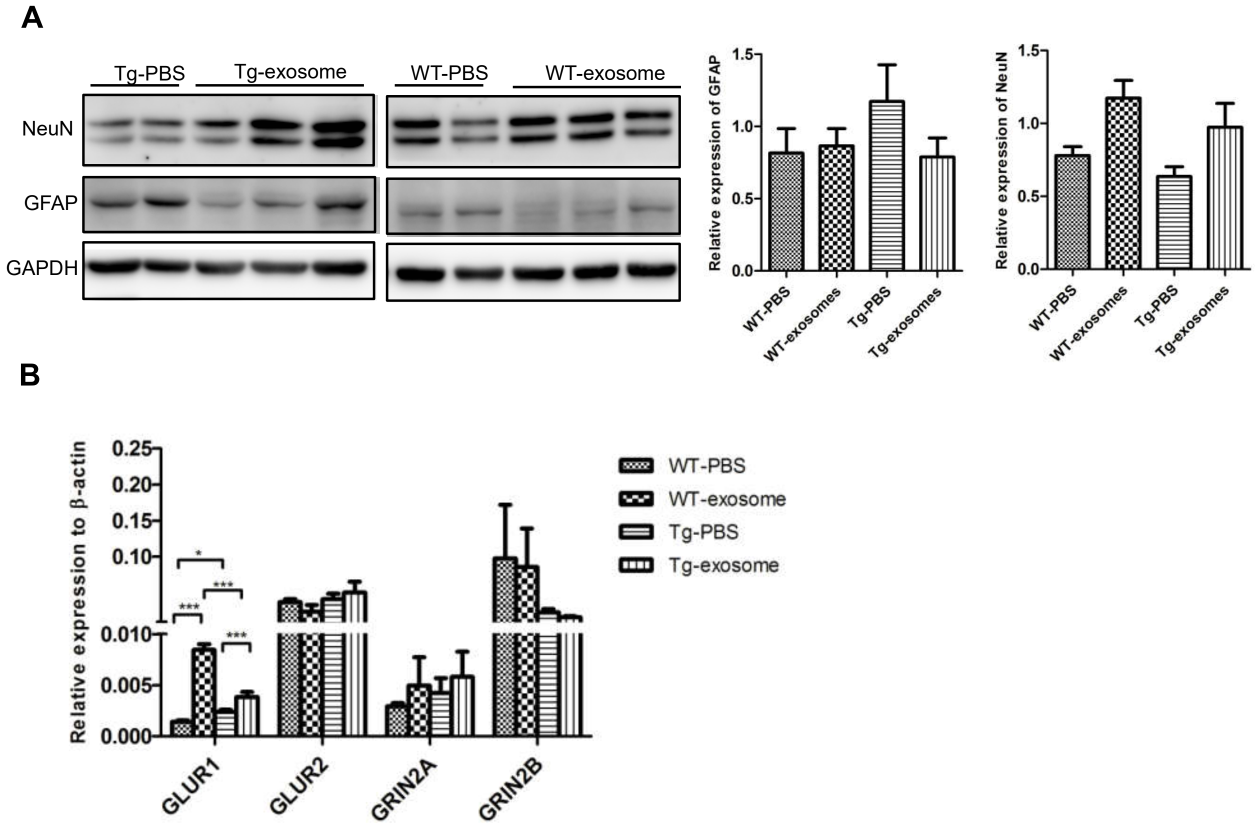

Figure S4. Effect of MSC-exosomes on neuron and AD related plasticity-related genes in Tg mice. (A) Western blot analysis of neuronal markers, NeuN and activated astrocyte marker, GFAP in MSC-exosomes/PBS treated WT and MSC-exosomes/PBS treated Tg mice. Right graph: the quantification of protein expression levels normalized against GAPDH levels in each sample (*P < 0.05; **P < 0.01; ***P < 0.001 by Tukey's *post hoc* test and showing nonsignificant among all groups). (B) Quantitative RT-PCR results of AD related affected genes from extracted hemispheres. Data are expressed as mean ± SEM (*$p < 0.05$ and ***$p < 0.005$ by Student's *t*-test).

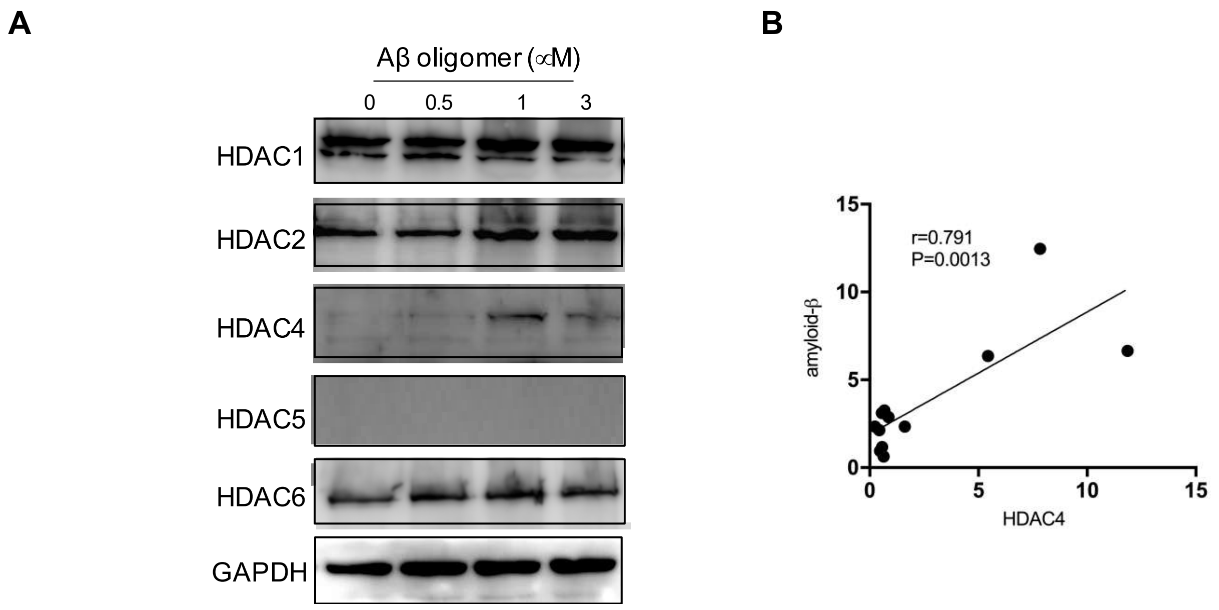

Figure S5. Aβ oligomers induce HDAC4. (A) Representative Western blots for HDAC4, other members of HDAC family and GAPDH loading control in neuroblastoma cell line SH-SY5Y in response to different dose of Aβ oligomers. (B) Pearson's correlation analysis was performed using GraphPad Prism software to calculate correlation coefficient. Scatter plot showing the correlation between HDAC4 and Aβ levels within FAD neural cell culture model.

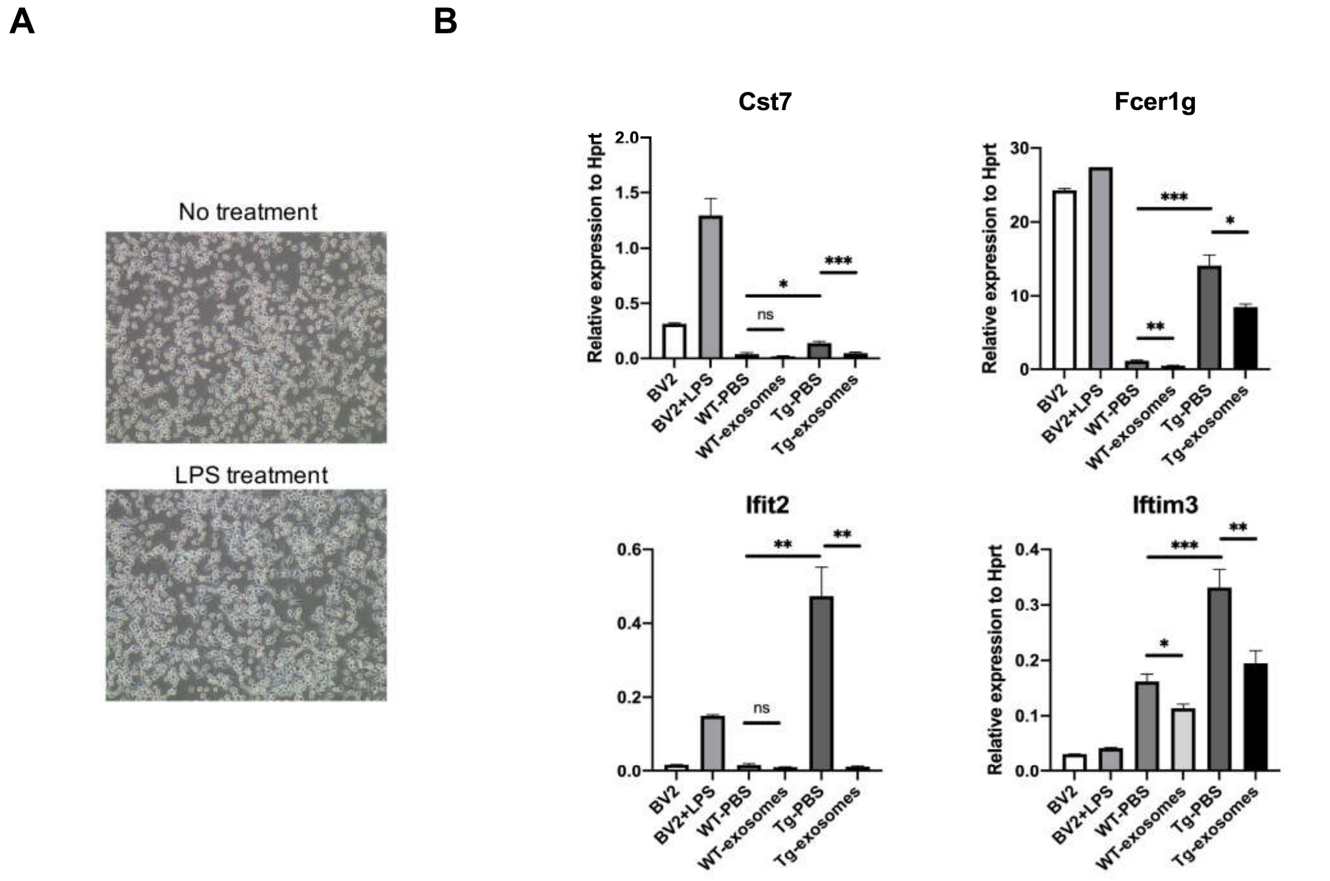

Figure S6. The effect of MSC-exosomes on activated microglial marker genes in Tg mice. Gene expression of LPS-treated BV2 cells (10 ng/ml) was used as positive control. Gene expression levels were normalized against *Hprt* levels (housekeeping gene) in each sample. Values are means ± SEM of triplicate well. 6 mice per each group. Statistics (WT-PBS versus Tg-PBS, WT-PBS versus WT-exosomes or Tg-PBS versus Tg-exosomes) were analyzed by unpaired Student's *t*-test. *p < 0.05, **p < 0.01, ***p < 0.001, ns, nonsignificant.

**Table S1:** Top 20 most highly expressed miRNAs in Human WJ-MSC derived exosomes		
**NO.**	**Mature miRNA**	**Read Counts**
**1**	hsa-miR-29a-3p	15,455
**2**	hsa-miR-125b-5p	7,096
**3**	hsa-miR-146a-5p	6,269
**4**	hsa-miR-16-5p	4,451
**5**	hsa-miR-199a-3p	4,120
**6**	hsa-miR-221-3p	3,736
**7**	hsa-let-7i-5p	2,858
**8**	hsa-miR-21-5p	2,739
**9**	hsa-miR-143-3p	2,564
**10**	hsa-let-7b-5p	2,540
**11**	hsa-miR-3135b	2,012
**12**	hsa-miR-7641	1,906
**13**	hsa-miR-382-5p	1,905
**14**	hsa-miR-122-5p	1,839
**15**	hsa-miR-134-5p	1,721
**16**	hsa-miR-510-3p	1,655
**17**	hsa-miR-432-5p	1,451
**18**	hsa-miR-409-3p	1,398
**19**	hsa-let-7a-5p	1,068
**20**	hsa-miR-1246	978

Table S1. Top 20 most highly expressed miRNAs in MSC exosomes according to NGS miRNA expression. NGS reads for a miRNA refers to the counts/million miRNA reads. Abbreviations: WJ-MSC, Wharton's jelly mesenchymal stem cell; miRNAs, microRNA; NGS, next generation sequencing

**Table S2**: memory/synaptic plasticity-related genes	
Gene name	Primers
Bdnf*IV*	Forward: 5'-CTCCGCCATGCAATTTCCAC-3'<br>Reverse: 5'-GCCTTCATGCAACCGAAGTA-3'
Synaptophysin	Forward: 5'-TGCGCTAGAGCATTCTGGG-3'<br>Reverse: 5'-CTTAAAGCCCTCGCCCCTTCT-3'
GLUR1	Forward: 5'-GTCTGGCTAACCCAGCTGTT-3'<br>Reverse: 5'-AAACCTTCAAACGCGACCT-3'
GLUR2	Forward: 5'-CTTTGTGCTCAACGTCAAGTTT-3'<br>Reverse: 5'-AAGATGTTGTAGCGGCCAAT-3'
GRIN2A<br>(NR2A)	Forward: 5'-TGGCCTCACCGGGTATGATT-3'<br>Reverse: 5'-CAATGCCGTCCCTCACTCTC-3'
GRIN2B<br>(NR2B)	Forward: 5'-GTCCCTGGACGATGGAGATTC-3'<br>Reverse: 5'-CAGTCAGCCCTACTGAGTTGG-3'
Homer1	Forward: 5'-CAACAGCTTGCTGCGTACC-3'<br>Reverse: 5'-CTAACACACTCCAGCTCAGTGAC-3'
Lgi1	Forward: 5'-CAAAGGCCTGGATTCCTTAAC-3'<br>Reverse: 5'-CCACAGTTGCATTGGTATGG-3'
SYN2	Forward: 5'-CCTTGGAGATTATGACATCAAGGT-3'<br>Reverse: 5'-GCCACCAGGTTAAGCTCGA-3'

Table S2. List of specific primers for memory/synaptic plasticity-related genes used in qPCR.

**Table S3**: activated microglia markers	
Gene name	Primers
Mouse Cst7	Forward: 5'- CCTGCCTTGAAGCGGACTC -3'<br>Reverse: 5'- CACCTCAAAACTGTGGAGCCA -3'
Mouse Fcer1g	Forward: 5'- CTGTCTACACGGGCCTGAAC -3'<br>Reverse: 5'- AAAGAATGCAGCCAAGCACG -3'
Mouse Ifit2	Forward: 5'- CTGAAGCTTGACGCGGTACA -3'<br>Reverse: 5'- ACTTGGGTCTTTCTTTAAGGCTTCT -3'
Mouse Ifitm3	Forward: 5'- CTCCATCCTTTGCCCTTCAG -3'<br>Reverse: 5'- TCTTCCTTGATTCTTTCGTAGTTTG -3'
Mouse Hprt<br>(internal CTL)	Forward: 5'- ATGGGAGGCCATCACATTGT -3'<br>Reverse: 5'- ATGTAATCCAGCAGGTCAGCAA -3'

Table S3. List of specific primers for activated microglia marker genes used in qPCR.

MATERIALS AND METHODS

Measurement of NEP-specific enzyme activity

Briefly, MSC-exosomes were incubated with the synthetic substrate (Abz-based peptide) (BioVision Inc., Milpitas, CA, USA) at 37°C to release a free fluorophore. Fluorescent intensity was detected using a fluorescence microplate reader at excitation and emission wavelengths of 330 nm and 430 nm, respectively, in kinetic mode for 60 minutes. The absolute NEP-specific activity was quantified using serial dilutions of recombinant human neprilysin (rhNEP) as a standard. The gradient of the time course of the fluorescent intensity in its linear region was determined at each concentration of the rhNEP dilution series to produce a standard curve. NEP activity was expressed in μU/mg protein. The calculation was demonstrated as follows: Sample NEP activity = B/(Δt x V) x D = pmol/min/ml = μU/ml, where B = Abz from standard curve (pmol), Δt = Reaction time (min.), V = Sample volume added into the reaction well (ml), D = Sample Dilution Factor. Unit Definition: One unit of NEP activity is the amount of enzyme that catalyses the release 1μmol Abz per min from the substrate under assay conditions at 37°C (pmol/min/mg=μU/mg).

Construction of lentivirus expression vector

To generate lentivirus expression vector carrying triple FAD mutations, we cloned full-length human β-amyloid precursor protein (APP_{695}) with the V717I (London) mutation from 3xTg AD mouse (JAX-34830). Next, to introduce Swedish double mutations K670N/M671L into the APP_{695} (London) gene, we performed a site-directed mutagenesis using two mutagenic primers, 5'-cggaggagatctctgaagtgaatttggatgcagaattccga-3' and 5'-tcggaattctgcatccaaattcacttcagagatctcctccg-3' by using the site-directed mutagenesis kit (Cat: 210518, QuikChange Lightning, Agilent, Santa Clara, USA). Then, APP (Swedish/London, S/L) cDNAs were cloned into lentiviral pLAS2.1w-EGFP-I2-puro vector to generate pLAS2.1w-APP_{695} (S/L)-EGFP. The primers, 5'-ctagctagctagatgatgcccggtttgg-3' and 5'-ccaatgcattggttcctagttctgcatctgctc-3' were used for cloning full length APP_{695} (S/L). The amplified APP_{695} (S/L) gene was double-digested with NheI and Nsil and ligated to pLAS2.1w-EGFP-I2-puro vector with flanking same cutting sites. All the newly constructed vectors were confirmed by sequencing (Missionbiotech, Taipei, Taiwan).

Generation of stable lines by lentivirus infection

We transfected the 293T cells with the pLKO-AS3-APP_{695} (S/L)-EGFP, pCMV-ΔR8.91 and pMD.G to produce lentivirus. 1ml viral solution was added to 85% confluent SH-SY5Y cells in 6-well plate, incubated overnight, and replaced growth media with 1μg/ml puromycin. The expression of the infected genes was confirmed by GFP expression by fluorescence microscopy. Resistant cell lines were analyzed by western blotting to confirm the Aβ overexpression.

Paraffin embedding and IHC staining of thick-layer FAD cultures

FAD thick layer cultures were fixed with 4% PFA at room temperature overnight for paraffin embedding. The PFA-fixed Matrigel was then embedded in paraffin, then cut in a microtome (Leica RM2235, Wetzlar, Germany) to 8μm thick sections and affixed onto the slides. For IHC staining, the slides were performed using a pressure cooker in sodium citrate buffer for 5 min and further incubated with the 1% BSA in TBS at room temperature for 1 hour to reduce nonspecific background staining. After incubation with the primary antibody against Aβ, p-Tau and Tuj-1 overnight at 4°C, the slides were incubated with 0.3% (v/v) H_2O_2 for 10 min at room temperature to block endogenous peroxidase activities and then incubated with HRP-conjugate secondary antibody for 1 hour and developed by EnVision Dual Link system DAB kit (DAKO, K4065, Agilent, Santa Clara, USA). The antibodies and dilution rates in this study were as follows: anti- Aβ antibody (1:100, 4G8, BioLegend, San Diego, CA, USA and 1:400, 6E10, Covance Inc, Princeton, NJ, USA); anti-pTau antibody (Ser396 and Ser404, 1:200, Arigo, Taiwan); anti-Tuj-1 antibody (1:500, ab18207, Abcam, Cambridge, UK); HRP-conjugate goat anti-mouse IgG (1:500, ab136815, Abcam, Cambridge, UK); HRP-conjugate rabbit anti-goat IgG (1:2000, ab6741, Abcam, Cambridge, UK); HRP-conjugate goat anti-rabbit IgG (1:500, ab6721, Abcam, Cambridge, UK).

Next-generation sequencing (NGS) analysis

To screen small RNA sequence comprehensively, especially microRNAs of WJ-MSC derived exosomes, RNA concentration was measured using a NanoDrop instrument (Thermo Fisher Scientific Inc., Waltham, MA, USA) and sent to Genomics Inc. (Taipei, Taiwan) for small-RNA sequencing. Briefly, exosomal RNA integrity was detected by Agilent Technologies 2100 Bioanalyzer (Agilent, Santa Clara, USA). Small-RNA libraries were prepared by adapter ligation, RT-PCR amplification and pooled-gel purification. The prepared libraries were quantified using qPCR according to the Illumina qPCR quantification protocol guide. Sample preparation and sequencing was conducted in duplicate.
